# Gap arthroplasty with active mouth opening exercises using an interocclusal splint in temporomandibular joint ankylosis patients

**DOI:** 10.1186/s40902-019-0200-x

**Published:** 2019-04-19

**Authors:** Min Woo Park, Mi Young Eo, Bo Yeon Seo, Truc Thi Hoang Nguyen, Soung Min Kim

**Affiliations:** 1 Department of Oral and Maxillofacial Surgery, Dental Research Institute, School of Dentistry, Seoul National University, 101 Daehak-ro, Jongno-gu, Seoul, 110-768 South Korea; 2 Department of Orthodontics, Dental Research Institute, School of Dentistry, Seoul National University, Seoul, South Korea

**Keywords:** Temporomandibular joint (TMJ) ankylosis, Gap arthroplasty, Interpositional graft, Interocclusal splint (IOS), Maximum mouth opening (MMO)

## Abstract

**Background:**

Temporomandibular joint (TMJ) ankylosis during early childhood may lead to disturbances in growth and facial asymmetry and to serious difficulties in eating as well as in breathing during sleep. The purpose of this study is to describe the effectiveness of an interocclusal splint (IOS) for active mouth opening exercises in the treatment of TMJ ankylosis.

**Methods:**

A total of nine patients with 13 instances of TMJ ankylosis from 2008 to 2010 were included in this study, of which five patients were male and four patients were female. Five patients demonstrated unilateral ankylosis, while five patients showed bilateral symptoms. Ankylosed mass resection with coronoidectomy, fibrotic scar release, and resection of stylohyoid ligament calcification was performed with gap arthroplasty without an interpositional graft, and all patients were assessed for maximum mouth opening (MMO) during a mean 6.6-year follow-up period.

**Results:**

All patients were subjected to postoperative mouth opening exercises from the day of the operation with the help of an IOS, which was based on an impression taken during surgery. All patients were sufficiently comfortable moving their mandible according to the IOS’s guiding plane and impingement, and satisfactory results were achieved, in which MMO was improved by 35 mm more than 6 years after surgery.

**Conclusions:**

Complete and adequate resection of the ankylosed mass and postoperative active mouth opening exercises are essential in the treatment of TMJ ankylosis. Moreover, a more comfortable mouth opening guide and interdigitation can be achieved using an IOS, and newly organized fibrosis in the gap space between the newly made resected condylar head and temporal fossa can be suggested.

## Background

Temporomandibular joint (TMJ) ankylosis might include fibrous or bony adhesions in the TMJ that limit functional opening of the mouth. The etiology of TMJ ankylosis can be idiopathic or related to congenital deformity, trauma, arthritis, infection, or previous TMJ surgery [1]. Localized or systemic infections, such as odontogenic origin or osteomyelitis of the long bones, are the most well-known reasons, and previous untreated mandibular condyle fracture including subcondylar or capping is also the frequent cause of TMJ ankylosis [2]. Previous surgical resection of TMJ involved tumors, or related radiotherapy could also induce fibrotic scarring with TMJ surrounding tissue indurations and, consequently, result in limited jaw motions. In addition, unknown autoimmune diseases such as systemic psoriasis, juvenile rheumatoid arthritis, or ankylosing spondylitis could also make TMJ ankylosis [2–6].

TMJ ankylosis is one of the disastrous and difficult diseases which change patients’ whole life styles including chewing, speech, and esthetic figurements. TMJ ankylosis during early childhood may lead to disturbances in growth and facial asymmetry and to serious difficulties in eating as well as in breathing during sleep [7, 8]. The final goal of TMJ ankylosis management will be re-establishment of mandibular movement and functions, facial appearance restoration, and normal growth in the child patients. Many operation skills with its outcomes have been represented and developed diverse techniques to decrease recurrences until recently. However, there are neither consensuses nor ideal treatment for TMJ ankylosis yet.

The purpose of this study is to provide a protocol for the use of an interocclusal splint (IOS) in the TMJ ankylosis patients. Complete resection of the ankylosed mass and postoperative active mouth opening exercises are essential for the treatment of TMJ ankylosis. Furthermore, a more comfortable mouth opening guide and interdigitation were achieved using an IOS. Based on these results, we emphasize the importance of early active physiotherapy and the use of an IOS in this clinical article.

## Methods

A total of nine patients with 13 instances of TMJ ankylosis were treated in the Department of Oral and Maxillofacial Surgery, Seoul National University Dental Hospital, from 2008 to 2010. Mean age is 35.4 years, and there were four females and five males. A unilateral case is in five, and bilateral in four patients. Causes of each patient are trauma in five, infection in two, and unknown in two patients. Preoperative assessment includes the patient’s photographs and interincisal maximum mouth opening (MMO), which ranged from 0 to 20 mm with an average initial MMO of 10.4 mm (Table 1). Three-dimensional (3D) computed tomography (CT) is essential for the initial diagnosis and rapid prototype model fabrication.

**Table 1:** Summary of clinical assessment and patient information used in this study

No	Age/sex	Etiology	Diagnosis	Operation	PreOp MMO (mm)	PostOp MMO (mm)	Follow-up and MMO	PostOp complications	IOS thickness (mm)
1	23/F	Trauma, re-ankylosis after 2 times attempts of gap arthroplasty and interpositional gap arthroplasty	Bilateral, right fibrous, Lt bony ankylosis	Both gap arthroplasty and both coronoidectomy	15	38	6 yrs, 43 mm	Anterior open bite	6
2	15/F	Unknown, born with prematurity re-ankylosis, one previous operation	Unilateral, Rt bony ankylosis	Rt, gap arthroplasty and both coronoidectomy	10	40	6.5 yrs, 35 mm	_	3
3	6/M	Trauma in childhood 3 yrs ago	Unilateral, Rt bony ankylosis	Rt, gap arthroplasty and both coronoidectomy	5	40	7 yrs, 38 mm	_	7
4	61/M	Trauma	Bilateral, bony ankylosis	Both gap arthroplasty and both coronoidectomy	0	35	7 yrs, 40 mm	_	4
5	15/F	Septicemia in childhood, re-ankylosis after 3 times attempts	Unilateral, Rt bony ankylosis	Rt gap arthroplasty and both coronoidectomy	3	43	6.5 yrs, 42.5 mm	_	3
6	62/F	Trauma (1 yr), zygomatico-maxillary fracture	Unilateral, Lt pseudoankylosis	Lt coronoidectomy and Lt arthrocentesis	13	40	6.5 yrs, 37 mm	_	3
7	65/M	Trauma (20 yrs), Rt facial skin defect	Scar condition and fibrosis of skin	Commissurorrhaphy and Lt coronoidectomy	20	35	6.5 yrs, 37 mm	_	5
8	41/M	Osteomyelitis in left mandibular ramus	Chronic osteomyelitis	Both coronoidectomy and Lt saucerization	14	37	7 yrs, 36 mm	_	5
9	31/M	Unknown, coronoid hyperplasia	Coronoid impingement on both sides, stylohyoid ligament calcification	Both coronoidectomy and both stylohyoidectomy	14	36	6.5 yrs, 48 mm	_	3

*MMO* maximal mouth opening between both incisal tooth tip or between alveolar ridge, *PreOp* preoperative, *PostOp* postoperative (in the operation room), *yr* year, *yrs* years, *Rt* right, *Lt* left, *IOS* interocclusal splint

Antibiotic prophylaxis was given preoperatively to all patients. Under the general anesthesia by fiberoptic endoscopic-assisted nasotracheal intubation, a preauricular or endaural skin incision was made in most patients, and the operation was performed according to the patient’s individual etiology. The main operation procedure included the following: (1) radical resection of ankylosed fused structure, (2) coronoidectomy on both sides, (3) arthrocentesis via arthroscopic instruments, (4) elongated calcified stylohyoid ligament resection via an intraoral approach, if needed, (5) additional saucerization in mandibular osteomyelitis patients, and (6) fibrotic scar release with radial forearm free flap reconstruction in severe facial scar patients. The postoperative MMO was checked directly in the operation room (Table 1). The radical resection or sufficient amount of resection was proceeded as complete resection of ankylosed bony structure not to be contacted in any mandibular movements, having a more than 2–3-mm interbony gap.

We used the IOS to improve the patient’s active mouth opening and closing exercises, and as a guide for the opening path and memorial exercises. After full mouth opening confirmation during operation, dental impressions of the maxilla and mandible were taken with polyvinyl President® (Coltene Co., Burlingame, Canada) rubber impression materials. Furthermore, bite registration using baseplate bite-wax sheets, at least 3.0 mm in thickness between incisal and molar cusps on both jaws, was also performed. In patient number 7, who had skin scar and fibrosis on his face, a 5.0-mm-thick bite registration was taken between both alveolar ridges due to loss of the anterior teeth. An IOS was created immediately with acrylic resin by conventional laboratory methods and delivered to the patient’s mouth while in the recovery room or general ward. With the help of several microscrews in the maxilla, the IOS was retained with a thin ligature wire on the labial side of the upper maxilla. Cognition of IOS and education for mouth opening and closing according to the intercuspation and guiding planes of the IOS were explained and checked at least once a day postoperatively. A maxillomandibular fixation or elastic traction was applicated according to each patient’s different situations, such as cooperative or difficulty of exercises. On the fixation period or elastic traction period, its duration is also dependent on each situation.

After discharge and within postoperative 10 days of surgery, all patients were instructed to sleep with the IOS and encouraged to do jaw opening exercises continuously according to the guide plane and interdigitation of IOS. The collection and processing of this retrospective clinical data was approved by the Institutional Review Board (IRB S-D20170029) of Seoul National University.

## Results

After surgical approaches to TMJ ankylosis treatment, each patient’s self MMO was measured as 35 to 43 mm, mean 38.2 mm in all nine patients. Active mouth opening exercises were begun on the day of operation with the help of an IOS. All patients were comfortable in mandibular active exercises according to the guiding plane and incisal tooth impingement of IOS.

**Fig. 1:**
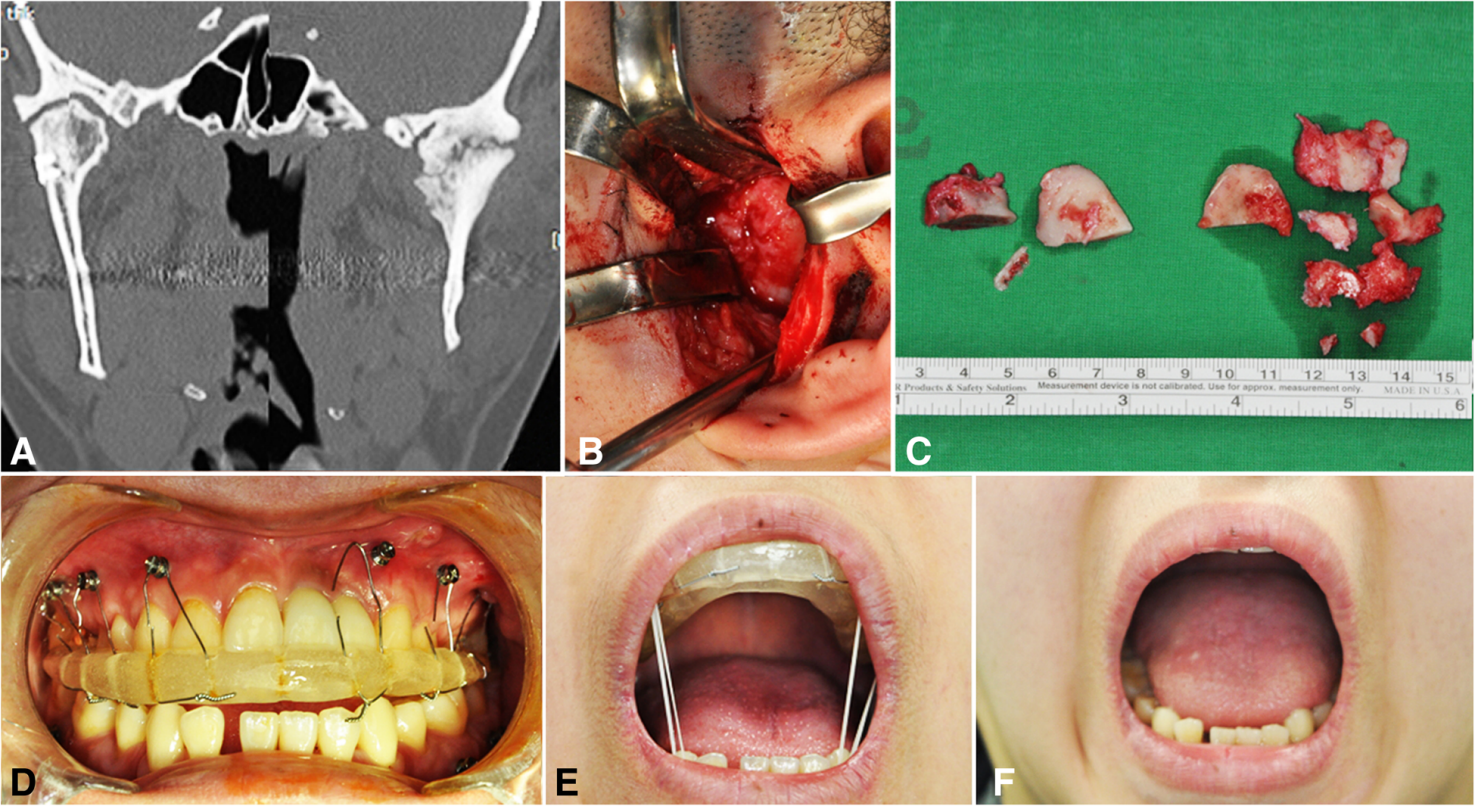
A 23-year-old female, bilateral TMJ ankylosis secondary to trauma, showing coronal CT scan (**a**), endaural approach with ankylosed mass resection at the left condyle (**b**), resected coronoid process and ankylosed bony mass from both sides (**c**), interocclusal splint retained on the maxillary screws (**d**), active mouth opening exercise with maximal interincisal opening (**e**), and mouth opening appearance after 6 years (**f**)

A 23-year-old female, patient number 1, underwent open reduction and fixation at the age of 18 because of bilateral condylar and symphyseal fracture of the mandible caused by a traffic accident. Over 5 years and 4 months, she underwent gap arthroplasty and interpositional arthroplasty on her left TMJ upon diagnosis of left TMJ ankylosis. Bilateral gap arthroplasty and coronoidectomy were performed in this clinical study, and with the help of IOS, the patient showed a MMO of 43 mm after 6 years (Fig. 1). The patient received additional orthodontic treatment for the correction of anterior open bite.

**Fig. 2:**
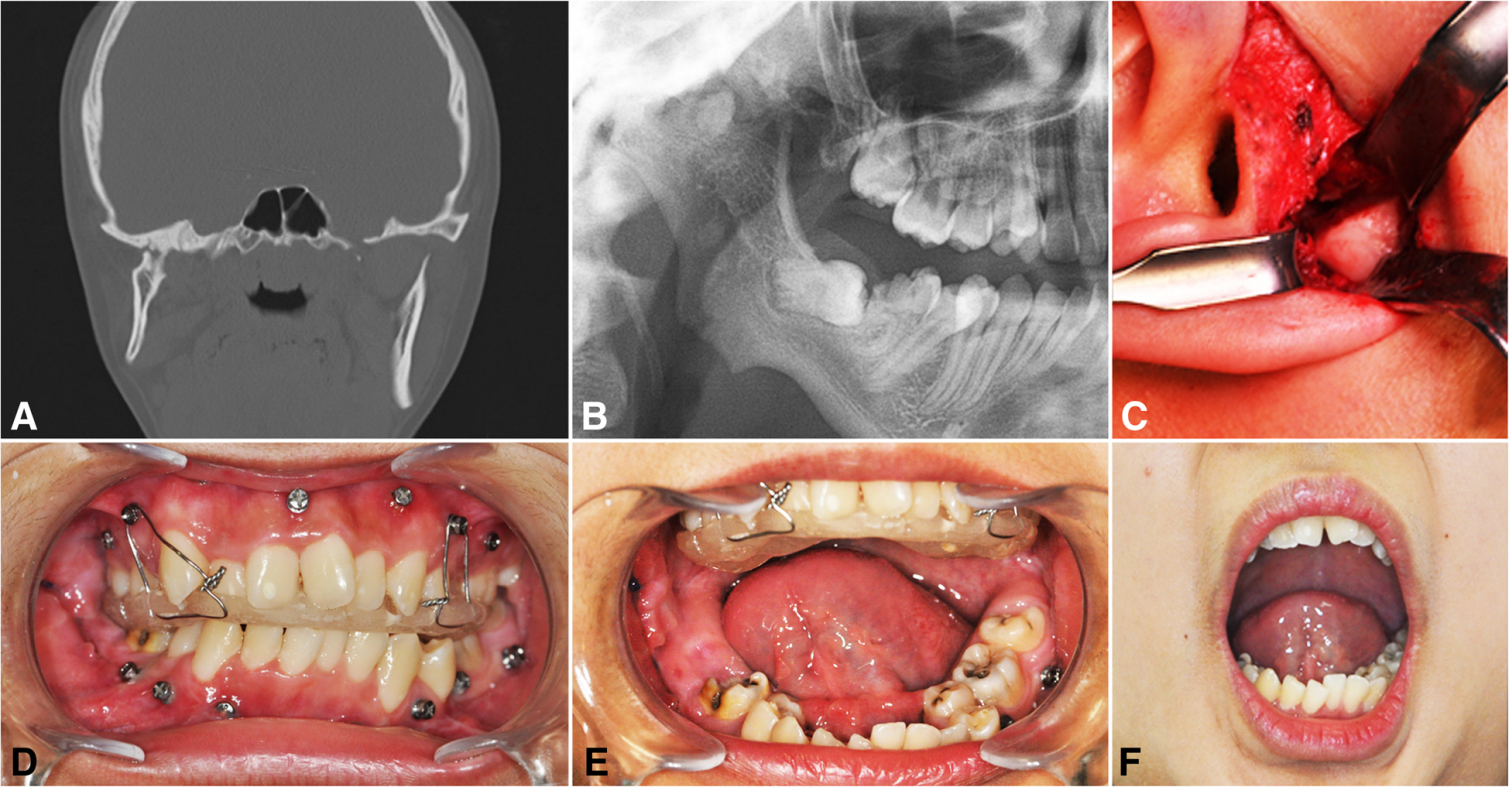
A 15-year-old female, idiopathic right side TMJ bony ankylosis, showing coronal CT scan (**a**), preoperative panoramic view (**b**), endaural approach with ankylosed mass resection (**c**), interocclusal splint retained on the maxillary screws (**d**), active mouth opening exercise with maximal interincisal opening (**e**), and mouth opening appearance after 6.5 years (**f**)

A 15-year-old female, patient number 2, underwent right TMJ surgery at the age of 6 due to mouth opening limitation, the etiology of which was unclear. Upon recurrence after her first surgery, right gap arthroplasty and bilateral condylectomy were performed by our surgical team. Preoperative MMO was 10 mm, which improved to 40 mm after operation. The patient was instructed to sleep with the 3-mm-thick IOS and was also encouraged to do jaw opening exercises during the daytime while wearing the IOS for occlusal stabilization. The patient showed very good results of 35 mm MMO after 6.5 years, and she underwent orthodontic treatment for the correction of tooth misalignment (Fig. 2).

**Fig. 3:**
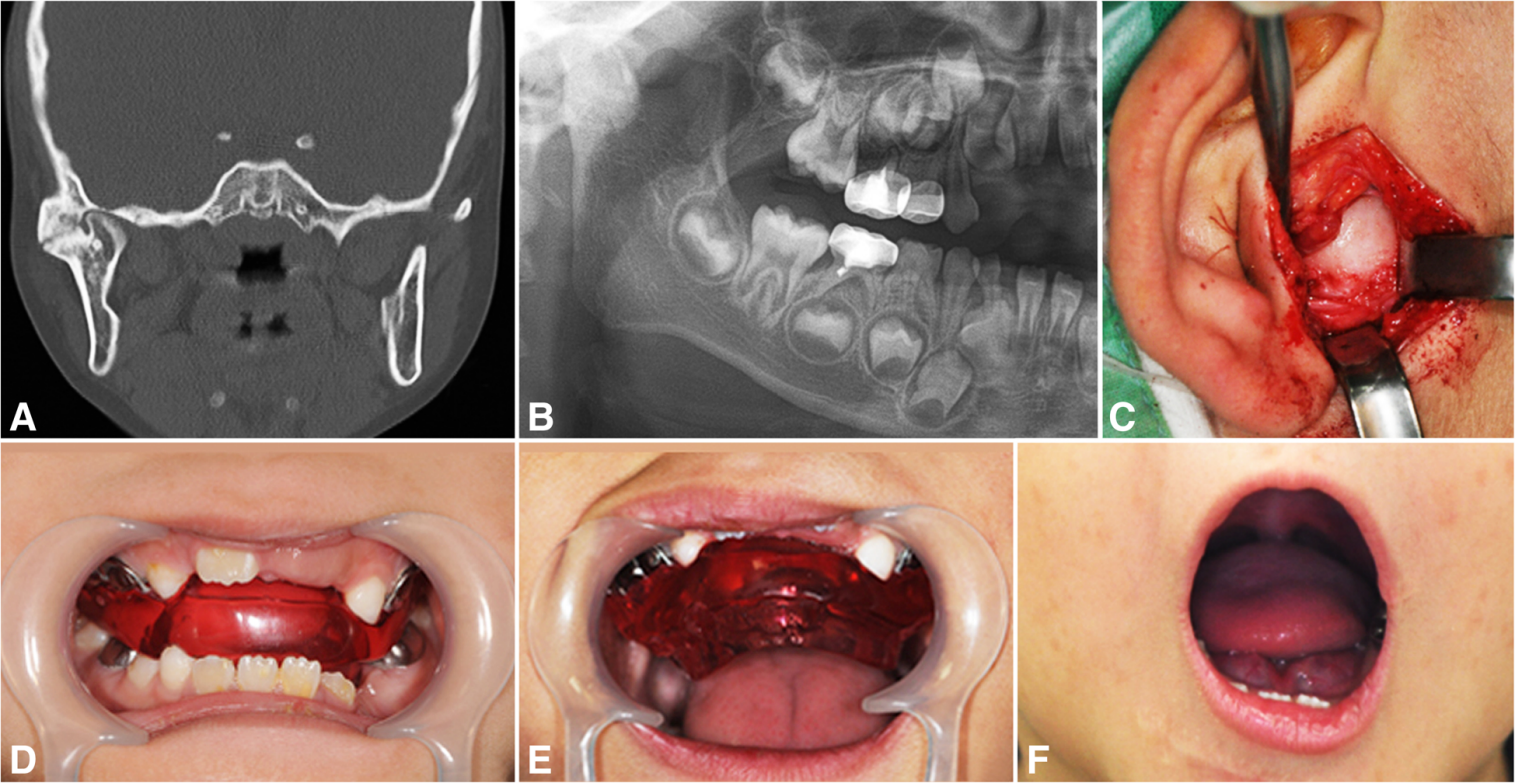
A 6-year-old boy, right side TMJ bony ankylosis secondary to trauma, showing right TMJ ankylosis with medial dislocation of fractured condyle in coronal CT scan (**a**), preoperative panoramic view (**b**), endaural approach (**c**), interocclusal splint retained on the upper teeth (**d**), active mouth opening exercise with maximal interincisal opening (**e**), and mouth opening appearance after 7 years (**f**)

A 6-year-old boy, patient number 3, had right TMJ ankylosis with a history of surgery at age of 3 due to bilateral condyle and symphysis fractures after a fall. Preoperative MMO was 5 mm, and after right gap arthroplasty and bilateral coronoidectomy, a postoperative MMO of 40 mm was attained. The patient was also instructed to sleep with the 7-mm-thick IOS and was encouraged to do jaw opening exercises during the daytime while wearing the IOS for occlusal stabilization (Fig. 3).

**Fig. 4:**
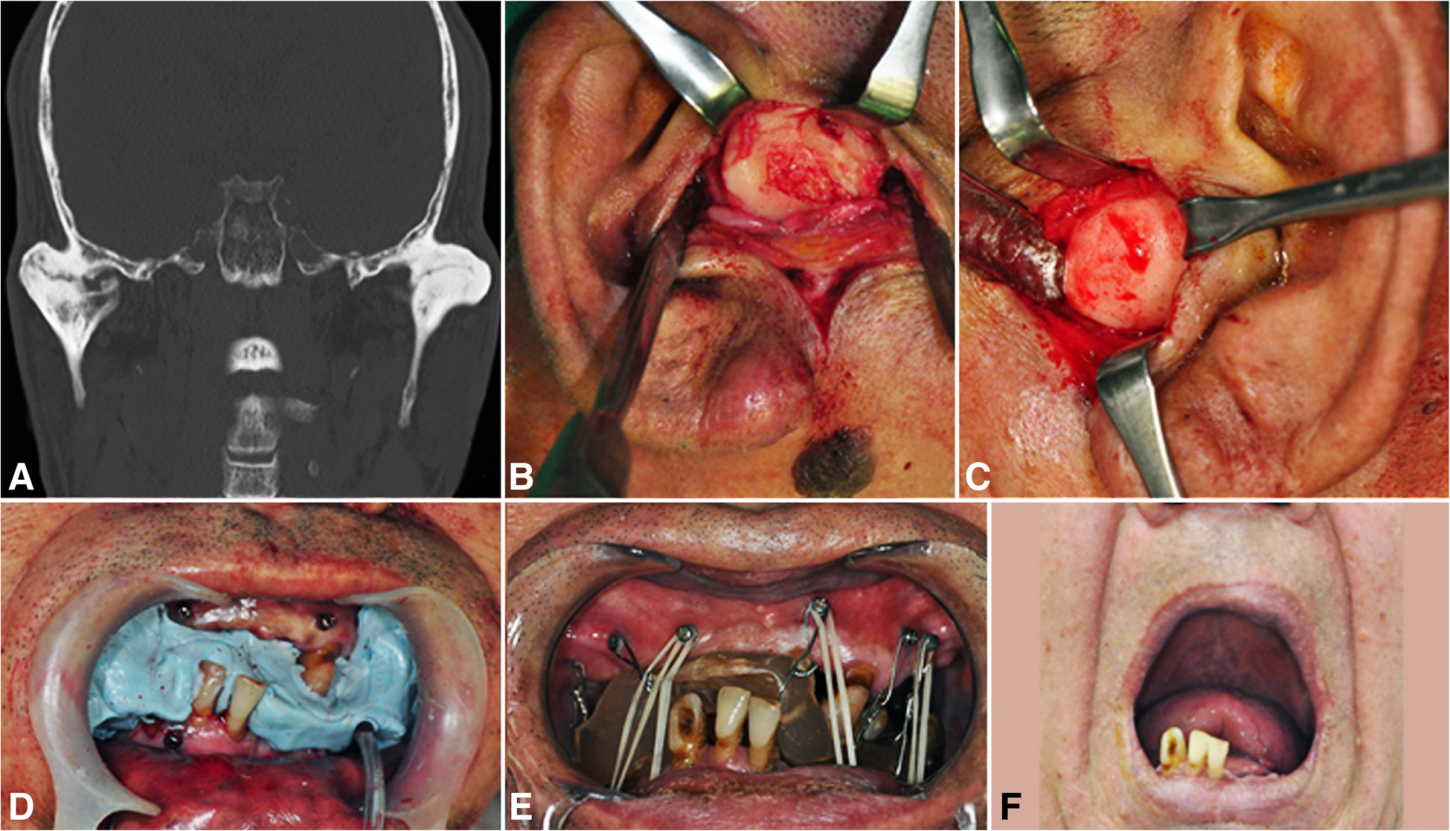
A 61-year-old male, bilateral TMJ ankylosis secondary to trauma, showing coronal CT scan (**a**), endaural approaches with ankylosed mass exposure at the right (**b**) and left (**c**) condyle, intraoperative bite registration to make an interocclusal splint (**d**), interocclusal splint retained on maxillary screws (**e**), and mouth opening appearance after active mouth opening on 7 years later (**f**)

A 61-year-old male, patient number 4, underwent operation for mandible fracture due to a fall that happened 27 years previous. Five years after surgery, he could not open his mouth; MMO was 0 mm. After bilateral gap arthroplasty with coronoidectomy, active physiotherapy was performed with the help of an ISO. MMO at discharge was 35 mm and improved to 40 mm after 7 years of follow-up (Fig. 4).

**Fig. 5:**
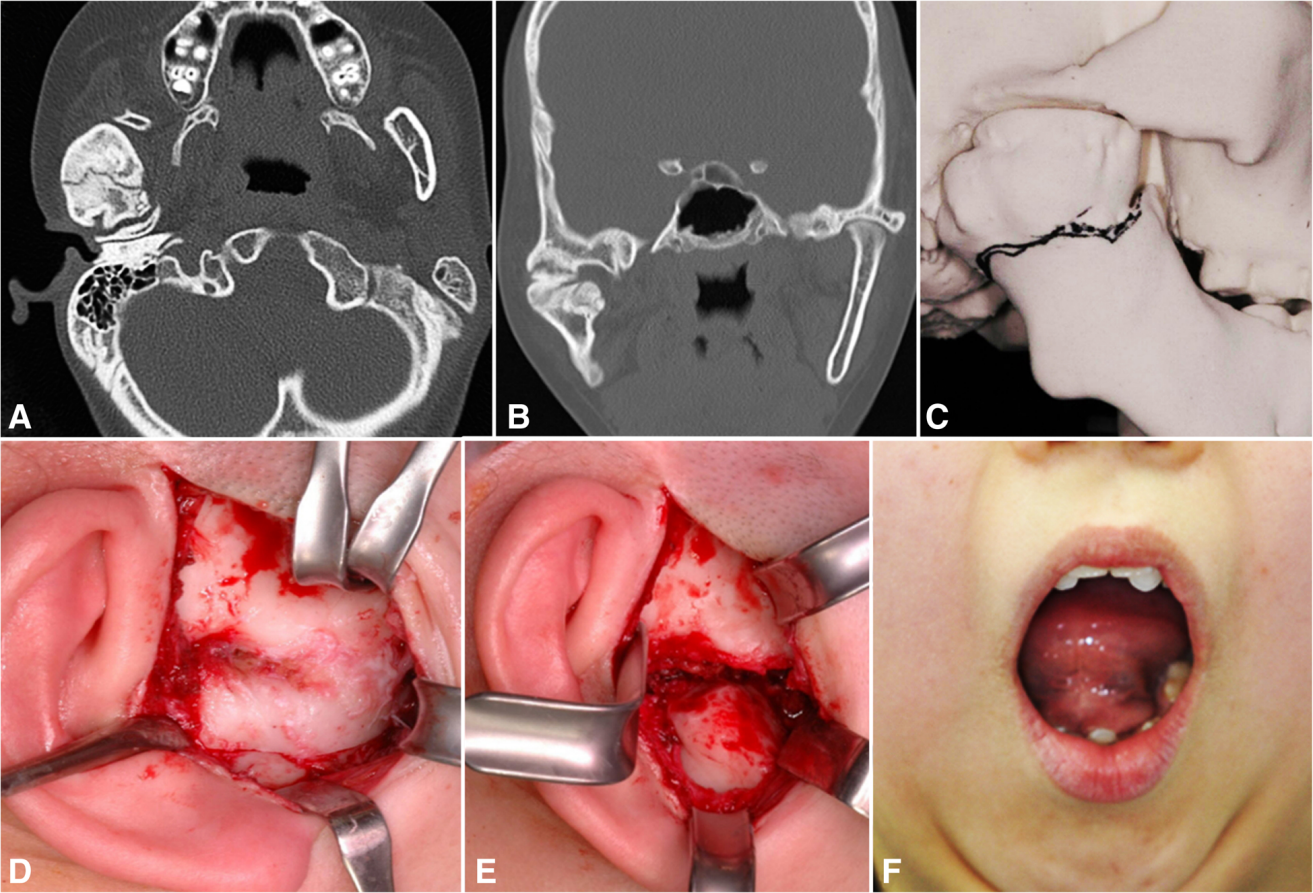
A 15-year-old female, right side TMJ bony ankylosis secondary to infection, showing axial (**a**) and coronal (**b**) CT scan, RP model used for surgical simulation in gap arthroplasty and coronoidectomy (**c**), endaural approach with ankylosed bony mass (**d**) and complete resection to the medical side of ankylosed mass (**e**), and mouth opening appearance after active mouth opening exercise with an interocclusal splint on 6.5 years later (**f**)

A 15-year-old female, patient number 5, experienced sepsis 1 year after her birth leading to difficulty opening her mouth. She underwent TMJ surgeries at the ages of 3, 5, and 9 years old. Preoperative MMO was 3 mm, and right gap arthroplasty with bilateral coronoidectomy yielded a postoperative MMO of up to 43 mm. After active mouth opening and closing exercises with the help of a 3-mm-thick IOS, a MMO of 42.5 mm was achieved at the 6.5-year follow-up (Fig. 5). The patient also underwent orthodontic treatment for the correction of tooth misalignment.

A 62-year-old female, patient number 6, had left TMJ pseudoankylosis, with a history of zygomatico-maxillary fracture due to trauma, which happened 1 year prior. Preoperative MMO was 13 mm. After left coronoidectomy with left arthrocentesis, active physiotherapy with the help of a 3-mm-thick ISO was performed. Postoperative MMO was 40 mm and was maintained at 37 mm throughout the 6.5-year follow-up period. A 65-year-old male, patient number 7, had scarring and fibrosis of the skin due to trauma that occurred 20 years prior. Preoperative MMO was 20 mm, and after commissurorrhaphy and left coronoidectomy, a 35-mm postoperative MMO was attained. A 41-year-old male, patient number 8, had osteomyelitis on his left mandibular ramus. Preoperative MMO was 14 mm, and after left ramus saucerization and bilateral coronoidectomy, a postoperative MMO of 37 mm was achieved. This patient showed a MMO of 36 mm after 7 years, with the help of a 5-mm-thick IOS. A 31-year-old male, patient number 9, had mouth opening limitation with a MMO of approximately 14 mm due to coronoid impingement on both sides, including stylohyoid ligament calcification. Both coronoidectomy and stylohyoidectomy were performed, and postoperative MMO was 36 mm. The patient was instructed to sleep with the 3-mm-thick IOS and was encouraged to do jaw opening exercises during the day with the IOS for occlusal stabilization. After active mouth opening and closing exercises with the help of a 3-mm-thick IOS, MMO was 48 mm at the 6.5-year follow-up.

**Fig. 6:**
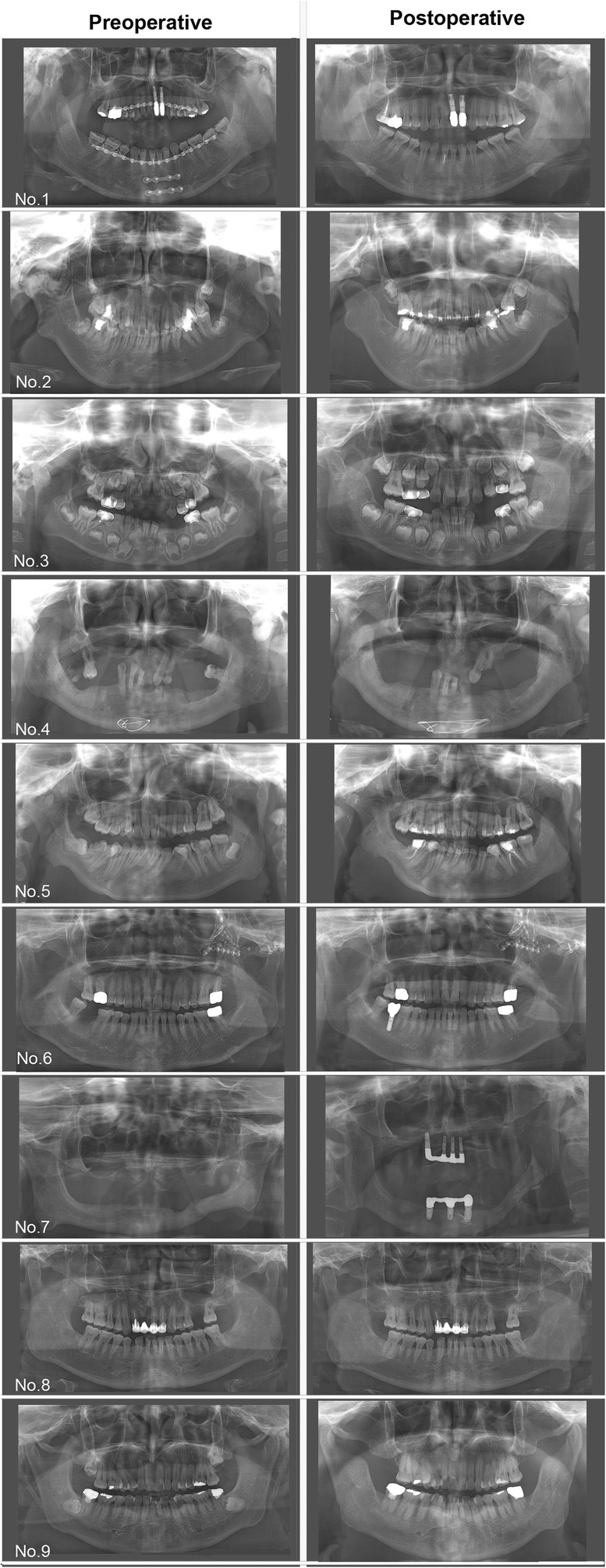
Preoperative and postoperative panoramic radiograms of nine patients according to each sequential number in Table 1. Each postoperative radiogram is taken on the final follow-up time as shown in Table 1

All patients have been followed and assessed during a mean period of 6.6 years. The mean MMO in the postoperative period was 38.2 mm. After more than 6 years of follow-up, one patient exhibited an anterior open bite, but no other recurrences or specific complications were observed. Marked improvement of mandibular movements with enhanced facial appearance was confirmed, and no re-ankylosis occurred in all patients (Table 1; Fig. 6).

## Discussion

Although diverse surgical techniques for TMJ ankylosis have been described by many expert surgeons, satisfactory restorations of mouth opening with acceptable functions are very difficult due to many unexpected factors. Thus, clinical studies and outcome evaluations of surgical techniques for TMJ ankylosis are important [8, 9]. In this clinical study, nine TMJ ankylosis patients with different etiologies were comfortable while moving their mandible according to their IOS’s guiding plane and impingement, and they showed satisfactory results, with MMO of more than 35 mm after a follow-up period of more than 6 years.

The choice of TMJ ankylosis management counts on many consideration factors including the patient’s age with growth spurts, systemic conditions related to previous infections or autonomic immune disease, and surgeon’s experiences for selection of interpositional grafting materials [9]. Simple excision or resection, gap arthroplasty with or without interposition materials, and whole joint replacement with autogenous or alloplastic substitutes are the frequently used surgical techniques. In spite of having the benefits of simplicity and short operation time, gap arthroplasty has some disadvantages, such as pseudo-articulation with a short ramus, removal failure of previous bony pathology, and a high risk of re-ankylosis. Moreover, it has also several potential complications, including postoperative open bite, deviated occlusion with posterior premature contact, and suboptimal mouth opening [8–11]. Recurrence or re-ankylosis is also the main complication of TMJ ankylosis management; thus, any interpositional substitutes such as autogenous temporal fascia or alloplastic silicone material at the joint resection site have been a basic concept of maintaining the interarticular space for the recurrence prevention. Until recently, several substances have been recommended as potential interpositional materials for placement into the surgically created interarticular gap, such as temporal myofascia. These interpositional materials create some fibrotic space to maintain sufficient space for the resected neo-condylar head to freely function during rotation and translation. However, some authors [2, 12] reported that a wider resected bony gap is more important than any interpositional substitutes used and most of re-ankylosis could occur due to incomplete resection of ankylosed bony or fibrotic tissues. Our focus in this clinical study was also on the radical resection of ankylotic tissues to prevent recurrence.

Danda and Chinnaswami [9] reported no significant different outcomes whether in interpositional arthroplasty or only gap arthroplasty in their 16 TMJ ankylosis cases, and Vasconcelos et al. [10] and Roychoudhury et al. [13] also reported good results of gap arthroplasty only each in their 8 cases and 50 cases, respectively. In our study, gap arthroplasty without an interpositional graft was performed in a total of nine patients, and we used an IOS for keeping the interarticular space instead of any interpositional substance with satisfactory results; the MMO was greater than 35 mm during the follow-up period (Table 1).

Previously, many surgeons used to neglect the importance of coronoidectomy in the TMJ ankylosis management, but coronoid process removal on both sides is one of the essential procedures due to the releasing effects of contracted temporal muscle and limited jaw movements [2]. If there is any lack of opening during operation, any slight fibrosis or contraction of the contralateral temporal muscle could be released by coronoidectomy together [13]. Thus, if a MMO value less than 25 mm is observed during operation, the contralateral joint and/or coronoid process with temporalis musculature should be released according to the nature of the opening limitation. We executed coronoidectomy in all our patients as the essential procedure for better results, ipsilateral in three patients and bilateral coronoidectomies in six patients (Table 1). Elongated stylohyoid ligament calcification can cause additional mouth opening limitation and severe neck pain. In our Eagle’s syndrome patient (patient number 9), both stylohyoidectomy with coronoidectomy were very helpful for mouth opening and normal mandibular movement [14]. For successful surgical results, at least 35-mm passive MMO should be acquired in the successful surgical results; therefore, complete resection of ankylosed mass with accompanying coronoidectomy is essential.

Immediate physiotherapy for TMJ patients is very important to prevent adhesion and subsequent re-ankylosis [12]. Roychoudhury et al. [13] showed the importance of postoperative physiotherapy for the prevention of re-ankylosis and for building muscular strength and bulk. Especially, increased mouth opening exercises should protract all the restricted masticatory muscles for re-adaptations of their previous inactive muscle tensions [15]. Furthermore, early mouth opening exercises could help the surgically established joint space become physiologically reorganized, so we used IOS to create and maintain this reorganized joint space.

**Fig. 7:**
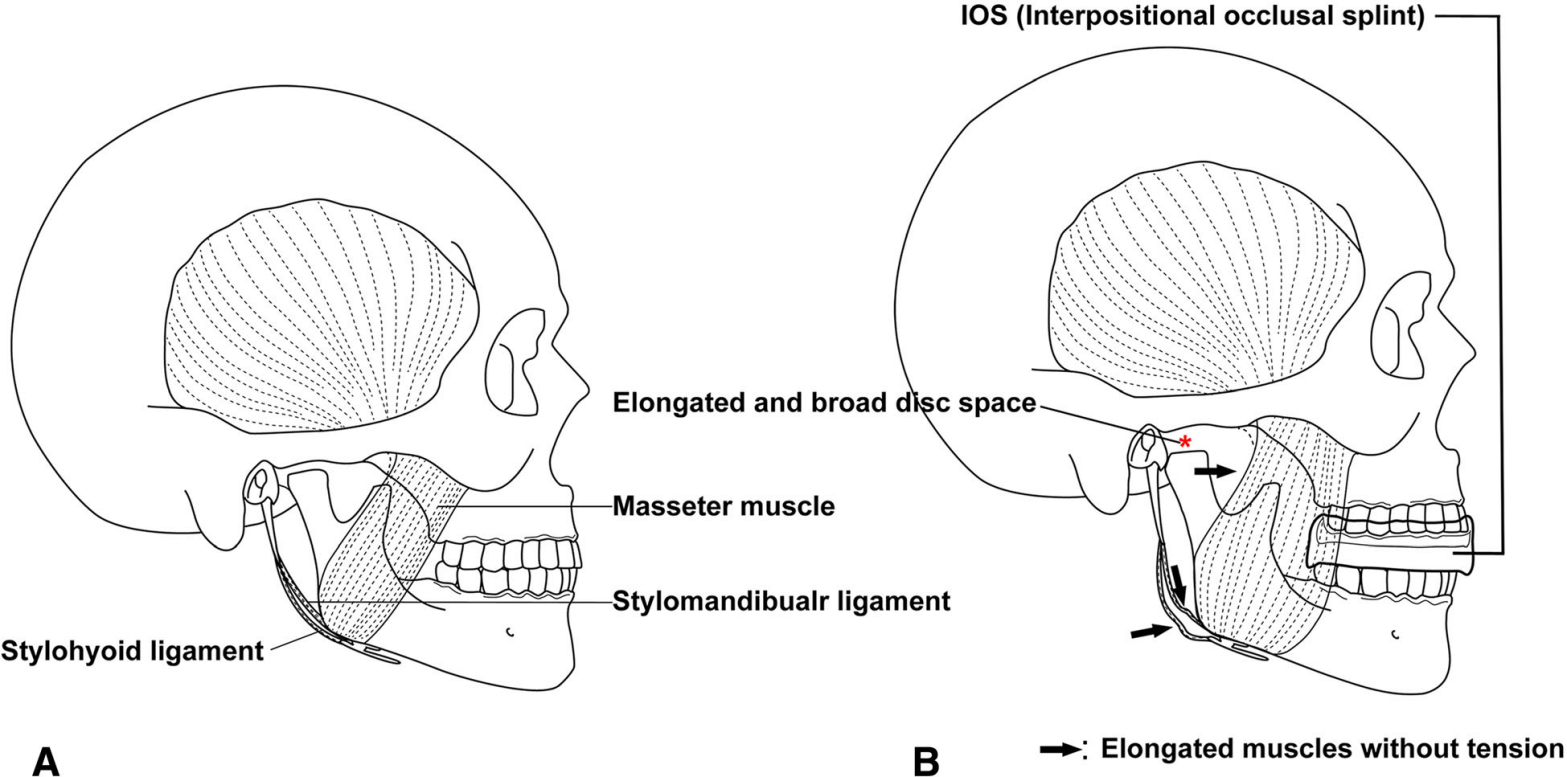
Schematic drawings of the basic actions of the interocclusal splint showing a tight articular disc space with the involved muscles without an IOS (**a**) and a loosened articular space with elongated or released muscles in IOS application (**b**)

We used IOSs to aide a patient’s active mouth opening exercises and to guide the opening path and memorial exercises as early as possible after operation and upon patient recovery from general anesthesia. Education regarding mouth opening and closing according to the intercuspation of IOS was continued at every follow-up in outclinic. In general, TMJ splint management could provide stability in his or her own occlusion to individual patient by offering a new position for the masticatory muscles and TMJ [16]. Compared with the conventional occlusal splint, this IOS could be thought as one of the thickened centric occlusion-guided splints. A thickness could be determined as postoperative mouth opening status of each patient, but at least 3.0 mm on both anterior and posterior teeth. Stable occlusion is essential for the adaptable position of the mandible and restoration of normal jaw functions by avoiding any neuromuscular imbalance in TMJ splint management. Therefore, IOSs used in physiotherapy after TMJ ankylosis operation relax previously contraction-related muscles and provide occlusal stabilization, thereby maintaining the stability of a newly organized gap in the TMJ (Fig. 7).

Mandibular opening induced by IOS thickness is accompanied by an increase in the joint space, and this space is expected to prevent any relapse potential. Ettlin et al. [17] reported that disc space could be increased on using a 3-mm-thick occlusal splint. By mouth closing and sliding mandible exercises with occlusal splint, a new oriented condyle-fossa relationship would be made without an intentional trial. Liu et al. [18] reported that the occlusal splint is useful in the mandibular condyle fracture child for good remodeling and keeping growth potentials. Additional protection disc space from secondary irritation could be acquired by shifting the condyle to lower location with the occlusal splint. Like these previous reports, IOS would change the condyle-fossa relationship, which could prevent re-attachment and help the resected surface of the condyle and fossa to be reorganized after surgical interventions. One patient showed the potential capacity of these splints to cause anterior open bite, as reported occasionally in the literature [19, 20]. Partially covering splints have been reported to induce occlusal modifications, including anterior open bite [21]. However, the patients in this study wore splints that covered all occlusal surfaces. Thus, these alterations are more likely due to positional changes in the condyle and fossa [22] as a possible consequence of the change in masticatory muscle activity [23], different distribution of occlusal load, or modifications in the vertical dimension [24, 25].

Consequently, postoperative active mouth opening and closing exercises are necessary for avoiding recurrent re-ankylosis. Furthermore, the use of an IOS is effective for guidance of comfortable mouth closing movements during active physiotherapy. After physiotherapy, new fibrous tissue might be formed at the gap space between resected condylar head and previously ankylosed temporal fossa through physiological adaptation. IOS acts as a guide for mouth opening exercises, enables more comfortable mandibular movement, and facilitates occlusal stabilization, thereby helping active postoperative physiotherapy.

## Conclusion

Gap arthroplasty without interpositional graft was performed in five patients, and other TMJ surgical approaches were used in four patients for the management of TMJ ankylosis. These approaches were evaluated in this clinical study. Complete evaluations of ankylosis etiology must be confirmed, and sufficient excision while maintaining a wide gap space, combined coronoidectomy, and other additional operative techniques must be executed according to individual cases. Thorough physical therapy with an IOS and periodic follow-up for more than 2 years after surgery are also necessary to avoid recurrence and complications. IOSs enable more comfortable mandibular movement and act as a guide for mouth opening exercises in active postoperative physiotherapy for TMJ ankylosis patients.

## Abbreviations

3D: Three-dimensional
CT: Computed tomography
IOS: Interocclusal splint
MMO: Maximum mouth opening
TMJ: Temporomandibular joint

## References

[1] Loveless TP, Bjornland T, Dodson TB, Keith DA (2010). Efficacy of temporomandibular joint ankylosis surgical treatment. J Oral Maxillofac Surg.

[2] Kaban LB, Bouchard C, Troulis MJ (2009). A protocol for management of temporomandibular joint ankylosis in children. J Oral Maxillofac Surg.

[3] Helenius LM, Tervahartiala P, Helenius I (2006). Clinical, radiographic and MRI findings of the temporomandibular joint in patients with different rheumatic diseases. Int J Oral Maxillofac Surg.

[4] Helenius LM, Hallikainen D, Helenius I (2005). Clinical and radiographic findings of the temporomandibular joint in patients with various rheumatic diseases. A case-control study. Oral Surg Oral Med Oral Pathol Oral Radiol Endod.

[5] Benazzou S, Maagoul R, Boulaadas M (2005). Ankylosis of the temporomandibular joint: a rare manifestation of ankylosing spondylitis. Rev Stomatol Chir Maxillofac.

[6] Kudryk WH, Baker GL, Percy JS (1985). Ankylosis of the temporomandibular joint from psoriatic arthritis. J Otolaryngol.

[7] Bulgannawar BA, Rai BD, Nair MA (2011). Use of temporalis fascia as an interpositional arthroplasty in temporomandibular joint ankylosis: analysis of 8 cases. J Oral Maxillofac Surg.

[8] Zhi K, Ren W, Zhou H (2009). Management of temporomandibular joint ankylosis: 11 years’ clinical experience. Oral Surg Oral Med Oral Pathol Oral Radiol Endod.

[9] Danda AK, S R, Chinnaswami R (2009). Comparison of gap arthroplasty with and without a temporalis muscle flap for the treatment of ankylosis. J Oral Maxillofac Surg.

[10] Vasconcelos BC, Bessa-Nogueira RV, Cypriano RV (2006). Treatment of temporomandibular joint ankylosis by gap arthroplasty. Med Oral Patol Oral Cir Bucal.

[11] Bayat M, Badri A, Moharamnejad N (2009). Treatment of temporomandibular joint ankylosis: gap and interpositional arthroplasty with temporalis muscle flap. Oral Maxillofac Surg.

[12] Kaban LB, Perrott DH, Fisher K (1990). A protocol for management of temporomandibular joint ankylosis. J Oral Maxdlofac Surg.

[13] Roychoudhury A, Parkash H, Trikha A (1999). Functional restoration by gap arthroplasty in temporomandibular joint ankylosis: a report of 50 cases. Oral Surg Oral Med Oral Pathol Oral Radiol Endod.

[14] Kim SM, Seo MH, Myoung H, Choi JY, Kim YS, Lee SK (2014). Osteogenetic changes in elongated styloid processes of Eagle syndrome patients. J Craniomaxillofac Surg.

[15] Dimitroulis G (2004). The interpositional dermis-fat graft in the management of temporomandibular joint ankylosis. Int J Oral Maxillofac Surg.

[16] Al-Ani Z, Gray RJ, Davies SJ (2005). Stabilization splint therapy for the treatment of temporomandibular myofascial pain: a systematic review. J Dent Educ.

[17] Ettlin DA, Mang H, Colombo V (2008). Stereometric assessment of TMJ space variation by occlusal splints. J Dent Res.

[18] Liu CK, Meng FW, Tan XY (2014). Clinical and radiological outcomes after treatment of sagittal fracture of mandibular condyle (SFMC) by using occlusal splint in children. Br J Oral Maxillofac Surg.

[19] Todd MA, Freer TJ (1994). Case report-anterior open bite as complication of splint therapy. Aust Orthod J.

[20] Nissani M (2001). A biblographical survey of bruxism with special emphasis on non-traditional treatment modalities. J Oral Sci.

[21] Uzun G, Keyf F (2011). Anterior open bite as a complication of the treatment of bruxism with anterior bite plane: a case report. Clin Dent Res.

[22] Fu AS, Metha NR, Forgione AG (2003). Maxillomandibular relationship in TMD patients before and after short-term flat bite plane therapy. J Craniomandib Pract.

[23] Dao TT, Lavigne GJ (1998). Oral splints: the crutches for temporomandibular disorders and bruxism?. Crit Rev Oral Biol Med.

[24] Demling A, Fauska K, Ismail F (2009). A comparison of change in condylar position in asymptomatic volunteers utilizing a stabilization and a pivot appliance. J Craniomandib Pract.

[25] Magdaleno F, Ginestal F (2010). Side effects of stabilization occlusal splints: a report of three cases and literature review. Cranio.

